# Template for Toxicants: Gene Expression Varies by Cell Type

**Published:** 2004-11

**Authors:** Julia R. Barrett

Gene expression profiling shows that cells generally respond to toxicant stress by repressing genes that guide cell growth and inducing those that govern DNA repair and other protective functions. However, the specific genes repressed or induced vary, depending on the cell type and—according to research presented in this issue—the toxicant to which the cells are exposed **[*EHP* 112:1607–1613]**. Melissa Troester of the University of North Carolina at Chapel Hill and colleagues note that this study demonstrates the utility of microarrays in predictive toxicology.

The current study builds upon previous research showing that separate breast cancer cell lines have distinctive responses to two different chemotherapeutic agents, doxorubicin (DOX) and 5-fluorouracil (5FU). Because DOX and 5FU have different mechanisms of action, the researchers hypothesized that cells treated with one compound would express a different transcription profile compared with cells treated with the other. In establishing support for this hypothesis, the researchers were also able to demonstrate that a profile of expressed genes could serve as a template to predict the mechanism of action for a third cancer drug, etoposide (ETOP).

The researchers cultured four breast cell lines for their experiments—two each of basal-like and luminal epithelium—and determined comparable toxic concentrations for DOX, 5FU, and ETOP at 36 hours’ exposure. Next, cell cultures were treated at these concentrations for 12, 24, or 36 hours in order to identify genes that were consistently expressed over time. At the end of the treatment periods, mRNA was extracted from the cells, pooled according to treatment and cell line, and used to create labeled complementary DNA samples. These samples were hybridized to microarrays representing 22,000 genes.

Microarray analysis identified which genes had been up- or down-regulated and revealed unique patterns of gene expression in response to DOX and 5FU in each cell type as well as each cell line. In general, luminal epithelial cells responded by regulating a large number of genes—974 in one line, 883 in the other. Basal-like epithelial cells regulated fewer genes (76 and 193) and also exhibited significant differences in gene expression over time. The cells exhibited a distinctly different profile at the 12-hour time point as compared with the 24- and 36-hour points. The difference was great enough that the DOX-treated samples clustered with 5FU-treated samples at 12 hours but not at 24 or 36 hours. This temporal shift blurred the lines between profiles and affected the accuracy of predictions.

Further investigation pinpointed 100 genes that could be used to differentiate between DOX- and 5FU-treated samples. This list of genes provided the basis for the final evaluation—testing whether the mechanism of action for ETOP could be accurately classified based upon the genes expressed following exposure. Because ETOP acts by a mechanism similar to that of DOX, it was expected that the gene set expressed by ETOP-treated cells would more closely resemble that of DOX-treated cells as compared to 5FU-treated cells.

Indeed, the mechanism of action for ETOP was predicted with 100% accuracy. When the researchers included cell type in the predictive model, the accuracy dropped to 75%, due in part to the temporal variability in gene expression in the basal-like cell lines.

With regard to the identity of regulated genes, published reports corroborate this toxicant-specific expression. For example, DOX has previously been shown to impair cellular respiration; the current research reveals that DOX alters mitochondrial gene expression, which provides a plausible explanation for the documented impairment. The findings also show several unanticipated changes in gene expression. For example, 5FU treatment induced the genes *ID1* and *ID3*, an effect that has not been previously noted. Knowledge of Id proteins is incomplete, and the researchers suggest that their pathways warrant attention as potential targets for therapeutic treatments.

Many toxicogenomics studies are providing expression data for toxicants that have known mechanisms of action, with the eventual goal of inferring mechanisms of action for novel compounds. Based on the success of their own mechanistic analysis, Troester and colleagues contend that this is feasible.

## Figures and Tables

**Figure:**
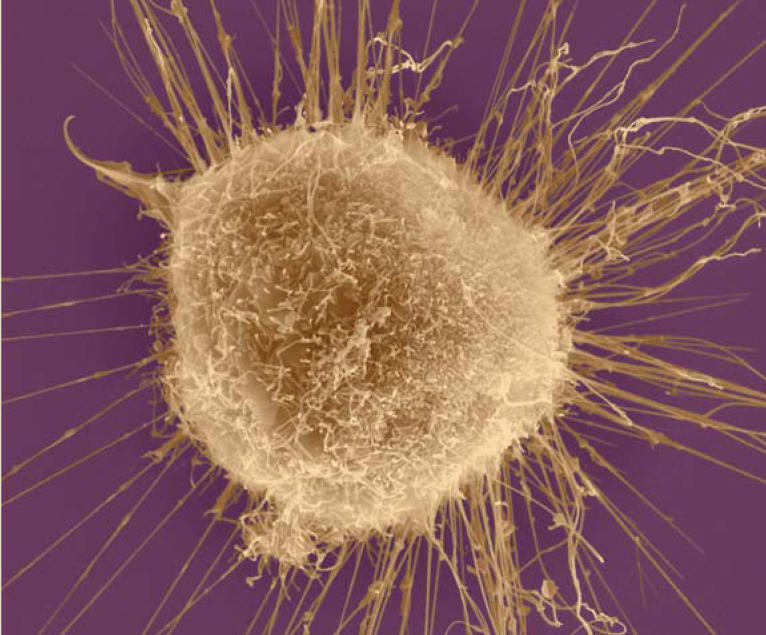
**Profiles in chemistry.** New research examining chemotherapeutic agents applied to breast cancer cells shows how known gene expression profiles may be used to predict the mechanism of action of other drugs.

